# The prevalence and factors associated with obesity and hypertension in university academic staff: a cross-sectional study in Bangladesh

**DOI:** 10.1038/s41598-023-34574-1

**Published:** 2023-05-05

**Authors:** Nurshad Ali, Shamim Ahmed, Shakil Mahmood, Aporajita Das Trisha, Firoz Mahmud

**Affiliations:** 1 Department of Biochemistry and Molecular Biology, Shahjalal University of Science and Technology, Sylhet, 3114 Bangladesh; 2 Department of Biochemistry and Molecular Biology, Gono University and Gonoshasthaya Samaj Vittik Medical College, Savar, Dhaka 1344 Bangladesh

**Keywords:** Cardiovascular diseases, Risk factors

## Abstract

Obesity is a major risk factor for hypertension, type 2 diabetes and other morbidities. On the other hand, hypertension is a leading cause of cardiovascular disease. The presence of obesity in hypertensive persons increases cardiovascular risk and related mortality. Data on the prevalence of obesity and hypertension in academic staff in Bangladesh are scarce. This study aimed to determine the prevalence and factors associated with obesity and hypertension among university academic staff in Bangladesh. In total, 352 academic staff were enrolled in this study from two universities in Bangladesh. A pre-structured questionnaire was used to obtain data on anthropometric, demographic and lifestyle-related factors. Bivariate and multivariate logistic regression analyses were performed to assess the factors associated with obesity and hypertension. Overall, the prevalence of general and abdominal obesity and hypertension was 26.7%, 46.9% and 33.7%, respectively. Female staff had a significantly higher prevalence of both general and abdominal obesity (41% and 64.1%, respectively) than male staff (21.5% and 34.9%, respectively) (*p* < 0.001). In contrast, male staff had a higher prevalence of hypertension (36.9%) than female staff (25.6%)(*p* < 0.001). An increased prevalence of hypertension was found in the higher BMI and WC groups of the participants. The prevalence of general obesity, abdominal obesity and hypertension was higher in the 30–40 years, > 50 years and 41–50 years age groups, respectively. According to the regression analysis, female gender and inadequate physical activity were independently associated with general and abdominal obesity. On the other hand, increased age, BMI, WC, presence of diabetes and smoking showed a significant association with hypertension. In conclusion, the prevalence of obesity and hypertension was higher among university academic staff members in Bangladesh. Our findings suggest that comprehensive screening programs are needed to facilitate the diagnosis, control, and prevention of obesity and hypertension in high-risk population groups.

## Introduction

Over the past few decades, the prevalence statistics on obesity have rapidly increased in developed and developing countries^1^. Based on the WHO report, 15% of women and 11% of men aged ≥ 18 years were globally obese in 2014^2^. Another form of obesity called abdominal obesity is a significant predictor of general obesity-related diseases^3^. It is well known that both general and abdominal obesity is associated with several metabolic disorders like hypertension, diabetes and cardiovascular disease^4^. On the other hand, hypertension is a leading cause of cardiovascular disease, accounting for 9.4 million global deaths per year^5,6^. A recent study that included data from 1990 to 2019 reported the global prevalence of hypertension as 32% in women and 34% in men^7^. According to the WHO report, the prevalence of age-standardized hypertension was higher in low-and middle-income countries compared to high-income countries^8^. In Asia, a large portion of the population lives in the South East Asian region and is facing a huge burden of hypertension and related mortality^9,10^. There is evidence that both obesity and hypertension are often found together^11^ which enhances cardiovascular risk and mortality^12,13^. A recent study indicated an intermediate to poor cardiovascular health metric score in three of every five adults in Bangladesh ^14^. Bangladesh is a lower-middle-income country in South East Asia, experiencing a rapid transition in demographic and socioeconomic status^15^. The rate of obesity and hypertension in this country has increased significantly over the last few decades. Some early studies showed a high prevalence of obesity and hypertension in the general population in Bangladesh^16–24^; however, there is no data for academic staff in Bangladesh. University academic staff members are one of the important groups of the national population. Moreover, a sedentary working environment and physiological stress among university academic staff may contribute to the development of obesity and hypertension. Therefore, there is a need to control obesity and hypertension in this important population group to prevent cardiovascular diseases and the serious consequences of hypertension. The purpose of this study was to determine the prevalence of obesity and hypertension and associated risk factors among university academic staff members in Bangladesh.

## Methodology

### Study participants and sampling

For this cross-sectional study, a total of 352 participants (male 241 and female 111) were recruited from the academic staff of two universities (Shahjalal University of Science and Technology, Sylhet and Gono University, Dhaka) in Bangladesh. This study was performed during the period between January 2018 and September 2019 at the Department of Biochemistry and Molecular Biology of Shahjalal University of Science and Technology, Bangladesh. The participants were selected using a simple random sampling technique. PASS version 15.0 was used for sample size calculation. A sample size of about 300 was needed to achieve 90% statistical power. The inclusion criteria were both genders and willingness to participate. As the exclusion criteria, lactating mothers, pregnant women, and participants who had a malignant disease were excluded during data collection. Participants missing any data were also not considered to include in the study. The Ethics Review Committee at the Biochemistry and Molecular Biology Department, School of Life Sciences, SUST approved this study protocol (ID 01/BMB/2018)). Written informed consent was obtained from all participants before the study commencement. All methods of the study were carried out in accordance with institutional guidelines and regulations.

### Data collection

A pre-tested questionnaire was used to collect data on anthropometrics (weight, height, body mass index, waist and hip circumference), demographic (age and sex), and lifestyle-related factors (physical activity and some food habits). The examinations and measurements were performed by two trained personnel. Anthropometric measurements were done according to the procedure described elsewhere^18,25–31^. The measurements were done with light clothing and without shoes. Body mass index (BMI) was calculated by dividing the weight in kilograms by the height in meter square (kg/m^2^). Blood pressure (BP) was measured with a digital BP machine (Omron M10, Tokyo, Japan). We advised the participants to take at least 10 min rest, then three consecutive BP measurements were taken 5 min apart. The first BP measurement was discarded and the average of 2nd and 3rd measurements was taken for systolic and diastolic blood pressures (SBP and DBP, respectively).

### Diagnostic criteria

The values of BMI were classified into underweight (< 18.5 kg/m^2^), normal (18.5–23.5 kg/m^2^), overweight (23.5–27.5 kg/m^2^) and obese (> 27.5 kg/m^2^) following guidelines suggested for the Asian population by WHO^32,33^. Abdominal obesity was diagnosed as a WC ≥ 80 cm for women and ≥ 90 cm for men^32,33^. Hypertension was diagnosed as SBP ≥ 140 mm Hg and/or, DBP ≥ 90 mm Hg and/or, current treatment for hypertension with antihypertensive drugs^34–37^. Prehypertension was diagnosed as SBP 120–139 mmHg; and/or DBP 80–89 mmHg^34–36^. Participants with diabetes were identified by checking prescriptions provided by physicians and/or self-reported use of anti-diabetic medications. Physical activity was defined using the Global Physical Activity Questionnaire (GPAQ) developed by the WHO^38^. Physical activity was categorized as low or sedentary, moderate, and adequate or vigorous. Smoking status was classified into nonsmoker and current smoker.

### Statistical analysis

Data are summarized as mean ± SD or percentages for the continuous and categorical variables, respectively. A chi-square test was used to assess the prevalence differences in the groups. Independent sample t-test and one-way ANOVA were used to measure the differences in the demographic and anthropometric variables. Bivariate and multivariable logistic regression analyses were performed to determine risk factors for hypertension and obesity. In regression models, the covariates such as age, sex, BMI, WC, presence of diabetes, physical activity, some food habits and smoking status were adjusted. The logistic regression results were expressed as odds ratio (OR) and 95% CI. Statistical data analyses were performed with the IBM SPSS Statistics version 23. A two-sided *p*-value < 0.05 was considered statistically significant.

## Results

### Characteristics of the study subjects

The demographic information of the study subjects by sex is summarized in Table 1. This study comprised 352 participants, 241 males and 111 females, aged 26–80 years. The mean age of the participants was 40.5 ± 10.3 years. The mean BMI was 25.7 ± 3.1 kg/m^2^ with significant deference between males (25.3 ± 2.6 kg/m^2^) and females (26.9 ± 3.9 kg/m^2^) subjects (*p* < 0.001). Males had a higher mean of WC (88.3 ± 6.7 cm) than the female (84.4 ± 9.4 cm) subjects (*p* < 0.01).

**Table 1:** Descriptive characteristics of the participants.

Measure	Total	Male	Female	*P*-value
*N*	352	241	111	–
Age (years)	40.5 ± 10.3	41.8 ± 10.4	37.3 ± 8.3	0.000
BMI (kg/m^2^)	25.7 ± 3.1	25.3 ± 2.6	26.9 ± 3.9	0.000
WC (cm)	86.7 ± 8.1	88.3 ± 6.7	84.4 ± 9.4	0.005
HC (cm)	94.9 ± 7.8	94.1 ± 5.6	96.1 ± 9.9	0.117
SBP (mm Hg)	121 ± 0.0	123.3 ± 13.1	115.2 ± 12.4	0.000
DBP (mm Hg)	82.1 ± 10.2	83.9 ± 9.6	77.7 ± 10.4	0.000
Diabetes				0.949
Yes (%)	21.9	21.8	22.1	
No (%)	78.1	78.2	77.9	
Intake of fat rich food				0.176
No (%)	39.5	39.6	29.7	
Occasionally (%)	18.3	15.3	25.0	
Yes (%)	45.2	45.1	45.3	
Intake of vegetables				0.853
Low (%)	8.7	8.3	9.4	
Medium (%)	66.8	68.1	64.1	
High (%)	24.5	23.6	26.6	
Intake of fruits				0.867
Low (%)	31.7	31.3	32.8	
Medium (%)	60.6	60.4	60.9	
High (%)	7.7	8.3	6.3	
Intake of raw salt				0.477
No (%)	81.7	81.3	82.8	
Yes (%)	18.3	18.8	17.2	
Physical activity				0.773
Low (%)	20.4	19.1	23.2	
Moderate (%)	71.0	72.4	68.1	
Adequate	8.6	8.7	8.6	
Smoking status	8.6	8.6	8.7	0.000
No (%)	89.3	85.0	100.0	
Yes (%)	10.7	15.0	0.0	

Data are presented as mean ± SD or percentage for continuous and categorical variables, respectively. WC: waist circumference, HC: hip circumference, BMI: body mass index, SBP: systolic blood pressure, DBP: diastolic blood pressure.

The overall mean SBP and DBP were 121 mm Hg and 82.1 ± 10.2 mm Hg, respectively, with a higher mean value in males than in females (*p* < 0.001). About 22% of the participants were diabetic. Our data showed that 45.2% of the participants were habituated to consuming fat-rich food. Only 8.6% of subjects were familiar with adequate physical activity and 10.7% were used to smoking.

### The prevalence of obesity and hypertension among the participants

**Figure 1:**
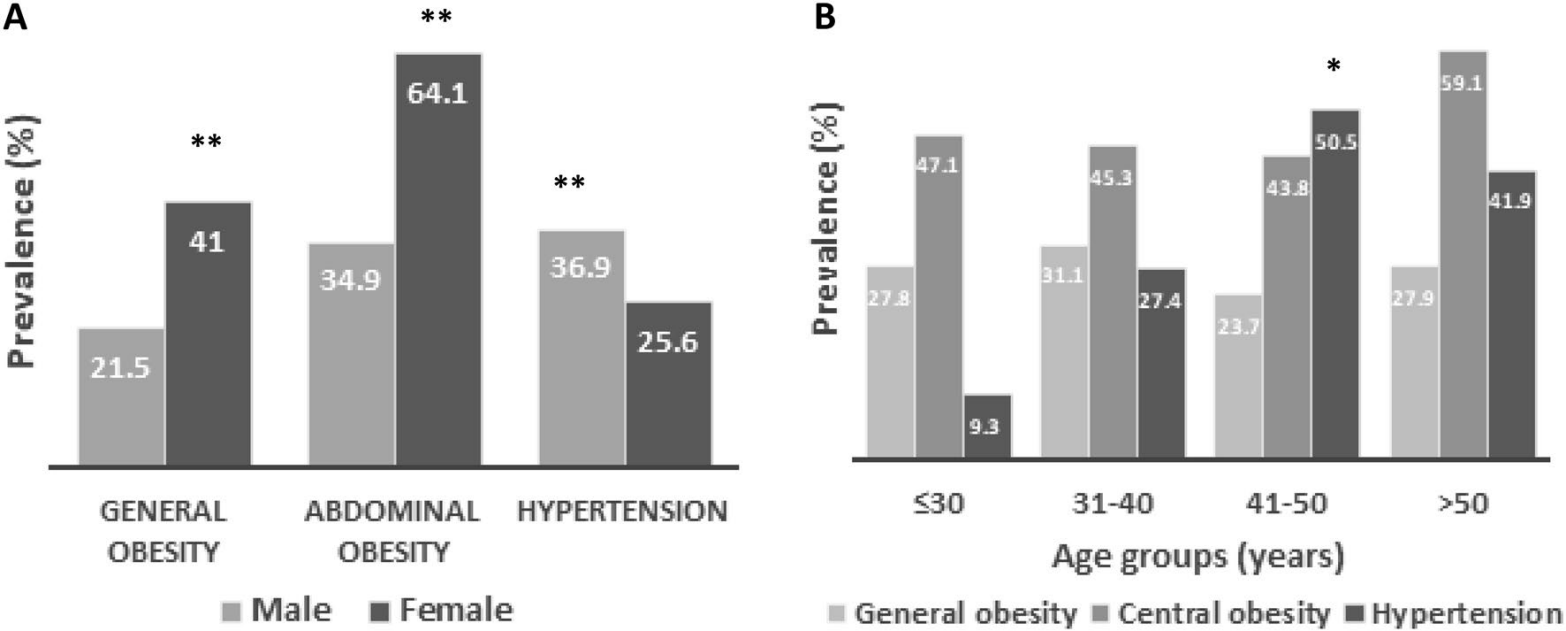
Prevalence of general and abdominal obesity and hypertension among participants by gender (**A**) and age groups (**B**). **P < 0.001 when the prevalence of hypertension is compared between the gender groups and *P < 0.01 when the prevalence is compared within the age groups. *P*-values are obtained from the chi-square test.

**Figure 2:**
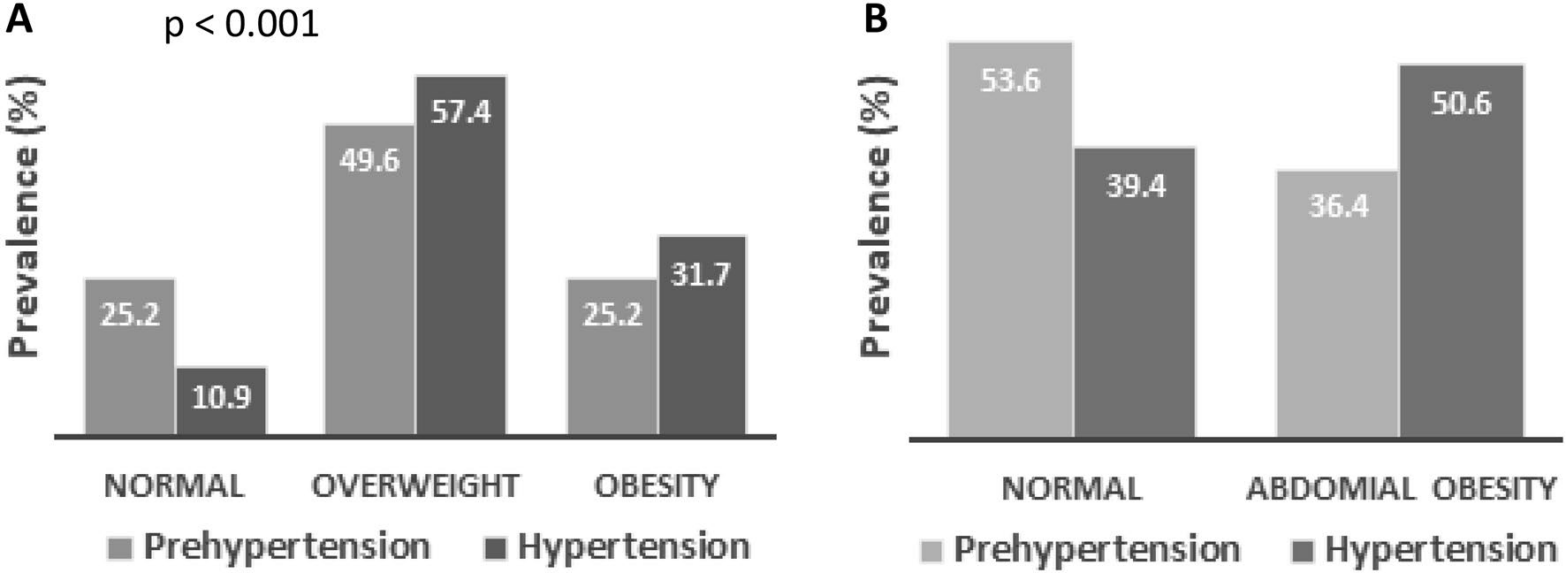
Prevalence of hypertension in the BMI (**A**) and WC groups (**B**). *P* < 0.001 when the prevalence of hypertension is compared between the BMI groups. *P*-values are obtained from the chi-square test.

The prevalence of obesity and hypertension are presented in Table 2 and Fig. 1. Overall, the prevalence of general and abdominal obesity was 26.7% and 46.9%, respectively. Overweight was prevalent in 51% of the study subjects. Both general and abdominal obesity was significantly higher in females (41% and 64.1%, respectively) than in males (21.5% and 34.9%, respectively) (*p* < 0.001). The prevalence rate of prehypertension and hypertension was 39.7% and 33.7%, respectively among the participants. Male subjects had a higher prevalence of prehypertension and hypertension (44.4% and 36.9%, respectively) than female (27.9% and 25.6%, respectively) subjects (*p* < 0.001). When participants were classified into four age groups, the prevalence of general obesity was higher in the 31–40 years age group, abdominal obesity was higher in the > 50 years age group and hypertension was higher in the 41–50 years age group. The prevalence of hypertension was significantly higher (*p* < 0.001) in overweight (57.4%) and obesity groups (31.7%) compared to the normal BMI (10.9%) group (Fig. 2). Similarly, hypertension prevalence was higher (50.6%) in the increased WC group compared to the normal WC (36.4%) group (Fig. 2) although the difference was not statistically significant.

**Table 2:** Characteristics of the study subjects according to BMI, WC and blood pressure data.

Gender	Body mass index			Waist circumference		Blood pressure		
	Normal (%)	Overweight (%)	Obesity (%)	Normal (%)	Obesity (%)	Normal (%)	Pre-hypertensive (%)	Hypertensive (%)
Male	22.4	56.1	21.5	65.1	34.9	18.7	44.4	36.9
Female	18.3	40.7	41.0	32.9	64.1	46.5	27.9	25.6
Total	21.7	51.6	26.7	53.1	46.9	26.7	39.7	33.7

Data are presented as percentages for the categorical variables. *P*-values indicate the differences in the prevalence between males and females. *P*-values are obtained from the chi-square test.

### Assessment of the risk factors by logistic regression analysis

Both bivariate and multivariate regression analyses were performed to determine the risk factors for obesity and hypertension. According to the analysis, female gender, increased WC, inadequate physical activity and low intake of vegetables were associated with the risk of general obesity (Table 3). In contrast, female gender, increased BMI and inadequate physical activity were the independent risk factors for abdominal obesity (Table 4). On the other hand, increased age, high BMI and WC, smoking and the presence of diabetes were the independent risk factors for hypertension among the study subjects (Table 5).

**Table 3:** Bivariate and multivariable logistic regression analysis to assess the factors associated with general obesity.

Variables	COR (95% Cl)	*P*-value	AOR (95% Cl)	*P*-value
Gender				
Male	Ref		Ref	
Female	2.76 (1.61–4.72)	0.000	2.69 (1.35–5.37)	0.005
Age (years)				
≤ 30	Ref		Ref	
31–40	1.18 (0.57–2.42)	0.662	1.78 (0.75–4.21)	0.192
41–50	0.81 (0.38–1.72)	0.581	1.01 (0.38–2.73)	0.981
> 50	1.01 (0.41–2.46)	0.989	0.81 (0.15–4.42)	0.807
WC (cm)				
Normal	Ref		Ref	
Obese	22.29 (7.95–62.51)	0.000	39.23 (8.66–177.72)	0.000
Intake of fat rich food				
No	Ref		Ref	
Occasionally	0.84 (0.33–2.17)	0.725	0.92 (0.33–2.59)	0.877
Yes	1.48 (0.75–2.94)	0.258	1.99 (0.92–4.32)	0.080
Intake of vegetables				
High	Ref		Ref	
Medium	2.69 (0.78–9.26)	0.117	3.20 (0.89–11.55)	0.076
Low	2.35 (1.02–5.43)	0.045	2.82 (1.18–6.77)	0.020
Intake of fruits				
High	Ref		Ref	
Medium	2.08 (0.42–10.22)	0.367	1.77 (0.34–9.13)	0.495
Low	2.81 (0.60–13.05)	0.188	2.52 (0.52–12.10)	0.249
Physical activity				
Adequate	Ref		Ref	
Medium	0.50 (0.16–1.54)	0.227	0.45 (0.13–1.56)	0.210
Low	1.30 (1.02–2.10)	0.044	1.10 (1.02–1.82)	0.046

COR: Crude odds ratio, AOR: Adjusted odds ratio, CI: Confidence Interval.

**Table 4:** Bivariate and multivariable logistic regression analysis to assess the factors associated with abdominal obesity.

Variables	COR (95% Cl)	*P*-value	AOR (95% Cl)	*P*-value
Gender				
Male	Ref		Ref	
Female	3.63 (1.80–7.33)	0.000	5.52 (2.04–14.97)	0.001
Age (years)				
≤ 30	Ref		Ref	
31–40	0.93 (0.43–2.01)	0.856	0.46 (0.15–1.40)	0.172
41–50	0.88 (0.28–2.71)	0.817	0.95 (0.22–4.06)	0.946
> 50	1.63 (0.59–4.47)	0.347	2.28 (0.59–8.80)	0.233
BMI (kg/m^2^)				
Normal	Ref		Ref	
Overweight	5.62 (1.22–25.91)	0.027	7.69 (1.53–38.71)	0.013
Obese	92.40 (16.56–515.52)	0.000	145.17 (22.11–953.05)	0.000
Intake of fat rich food				
No	Ref		Ref	
Occasionally	0.69 (0.22–2.14)	0.523	1.57 (0.30–8.26)	0.593
Yes	0.77 (0.30–1.97)	0.585	0.82 (0.20–3.45)	0.786
Intake of vegetables				
High	Ref		Ref	
Medium	1.89 (0.41–8.61)	0.413	2.70 (0.18–39.41)	0.469
Low	1.27 (0.44–3.69)	0.655	0.56 (0.09–3.57)	0.536
Intake of fruits				
High	Ref		Ref	
Medium	1.83 (0.17–19.31)	0.617	3.53 (0.10–122.88)	0.486
Low	3.11 (0.30–31.79)	0.338	6.83 (0.16–301.21)	0.320
Physical activity				
Adequate	Ref		Ref	
Medium	0.29 (0.07–1.22)	0.092	0.13 (0.01–1.61)	0.111
Low	1.50 (1.12–2.11)	0.042	1.56 (1.02–2.26)	0.048

COR: Crude odds ratio, AOR: Adjusted odds ratio, CI: Confidence Interval.

**Table 5:** Bivariate and multivariable logistic regression analysis to assess the factors associated with hypertension.

Variables	COR (95% Cl)	*P*-value	AOR (95% Cl)	*P*-value
Gender				
Female	Ref		Ref	
Male	1.70 (0.97–2.98)	0.062	1.44 (0.76–2.76)	0.267
Age (years)				
≤ 30	Ref		Ref	
31–40	3.69 (1.34–10.18)	0.012	3.08 (1.08–8.75	0.035
41–50	10.01 (3.67–27.26)	0.000	8.03 (2.86–22.53)	0.000
> 50	7.06 (2.25–21.23)	0.001	4.27 (1.35–13.52)	0.013
WC (cm)				
Normal	Ref		Ref	
Obese	1.46 (0.63–3.40)	0.377	1.48 (0.66–3.52)	0.470
BMI (kg/m^2^)				
Normal	Ref		Ref	
Overweight	2.77 (1.34–5.74)	0.006	3.09 (1.40–6.79)	0.005
Obese	2.91 (1.32–6.93)	0.008	3.94 (1.64–9.47)	0.002
Diabetes				
No	Ref		Ref	
Yes	2.87 (1.64–5.04)	0.000	2.35 (1.27–4.33)	0.006
Intake of fat rich food				
No	Ref		Ref	
Occasionally	0.82 (0.29–2.33)	0.705	1.09 (0.34–3.49)	0.886
Yes	1.58 (0.75–3.31)	0.226	1.45 (0.63–3.34)	0.378
Intake of vegetables				
High	Ref		Ref	
Medium	0.82 (0.20–3.39)	0.784	0.81 (0.16–4.03)	0.796
Low	1.24 (0.56–2.75)	0.600	1.05 (0.43–2.59)	0.914
Intake of fruits				
High	Ref		Ref	
Medium	0.44 (0.13–1.54)	0.201	0.68 (0.17–2.77)	0.590
Low	0.57 (0.18–1.81)	0.341	0.64 (0.18–2.33)	0.500
Intake of raw salt				
No	Ref		Ref	
Yes	0.49 (0.18–1.34)	0.163	0.56 (0.18–1.67)	0.296
Physical activity				
Adequate	Ref		Ref	
Medium	2.13 (0.41–10.92)	0.367	1.00 (0.16–6.26)	0.999
Low	3.67 (0.81–16.50)	0.091	1.70 (0.31–9.23)	0.538
Smoking				
No	Ref		Ref	
Yes	3.26 (1.54–6.92)	0.002	2.29 (1.03–5.08)	0.041

COR: Crude odds ratio, AOR: Adjusted odds ratio, CI: Confidence Interval.

## Discussion

The present study determined the prevalence and factors associated with obesity and hypertension in academic staff in Bangladesh. To the best of our knowledge, this is the first data on the prevalence of obesity and hypertension among academic staff members in Bangladesh. In our study, the total prevalence of general and abdominal obesity and hypertension was 26.7%, 46.9% and 33.7%, respectively.

In the present study, females had a higher prevalence of both general and abdominal obesity (41% and 64.1%, respectively) than males (21.5% and 34.9%, respectively). Similar findings were reported in a recent study that included both rural and urban adults from all divisional regions in Bangladesh^20^. In that study, the prevalence of general and abdominal obesity was 25.5% and 56.1% in females and 12.2% and 29% in male participants, respectively^20^ which are also slightly lower than the prevalence rate found in the present study. Another recent study in Bangladesh reported an increased prevalence of overweight/obesity in females (45.6%) than in males (32.7%)^39^. A higher prevalence of both types of obesity was also reported among female participants in other studies performed in India^40^ and China^41^. An increased prevalence of obesity in females might be related to excess calorie intake and low physical activity. Furthermore, using oral contraceptive pills, menopause and increased parity may also contribute to the development of obesity in females^42^.

In our study, most of the participants were used to living in urban or suburban areas. In Bangladesh, a significant portion of the urban inhabitants is involved in comfortable office-related work. In addition, urban people are used to consuming a healthier and fat contained diet but do less physical exercise which may also influence excess weight gain among them. An increased prevalence of abdominal and general obesity was found in urban participants than in rural participants in a previous study in Bangladesh^20,43^. A higher prevalence of obesity was also reported in urban people in Mayanmar^44^ and India^45^. It is important to mention that, a sedentary working environment and psychological stress may also increase the risk of obesity. A recent study reported that about 89% of university staff had moderate/high stress and only 25% of staff slept at least 8 h nightly^46^. There is also evidence that chronic stress may increase the risk of obesity as well as diabetes, hypertension, and cardiovascular disease^47^. Thus, a sedentary working environment and work-related stress may also contribute to the increased prevalence of obesity among our participants.

In our study, the participants had about twofold higher rates of abdominal obesity compared to the general obesity which suggests that a portion of the participants maybe were not diagnosed as obese based on their BMI values. Therefore, particular BMI cut-off values for both genders might be not sufficient to measure general obesity. In this case, age, gender and ethnic-specific BMI cut-off values may be more accurate for the diagnosis of general obesity. According to regression analysis, female gender, and insufficient physical activity were important risk factors for both types of obesity. Female gender and inadequate physical activity were also identified as the risk factors for obesity in the South Asian population^20,48^.

Some previous studies reported hypertension prevalence in the Bangladeshi general population; although, a variation in prevalence rate has been observed between the studies. An early review indicated the hypertension prevalence as 13.5% in Bangladeshi adults^49^. Another study reported the prevalence of hypertension at 27.4% in Bangladeshi adults^22^. A recent study that included data from rural and urban adults of eight divisional regions in Bangladesh reported the prevalence of hypertension at 30.9%^20^. The overall prevalence of hypertension in the present study (33.7%) is higher than in several previous studies including a very recent study conducted across the country (30.9%)^20^. Among our study subjects, the prevalence rate of hypertension was significantly higher in males (36.9%) than in females (25.6%). A study conducted in the neighbouring country Pakistan also reported a higher prevalence of hypertension among university male staff (33.6%) than in the female staff (27.6%)^50^. An increased prevalence of hypertension was also reported among male staff (58.1%) than in the female staff (41.9%) of a university in Saudi Arabia^51^. Another study conducted among university staff in Malaysia also reported an increased prevalence of hypertension in male staff (45.5%) than in female staff (22.9%)^52^. In our study, increased age, increased WC and BMI, the presence of diabetes and smoking were the significant risk factors for hypertension. Similar results were found in other studies conducted in Bangladesh and other Asian countries^20,43,53^. Among the identified risk factors, the unmodifiable factor is age^54^; therefore, in comprehensive programs, more attention needs to pay to the modifiable factors such as decreasing BMI, avoiding fatty food and smoking, doing regular physical exercise and control of diabetes^17,20,55^.

In our study, we observed a higher rate of hypertension in overweight and obese groups. There is evidence that obesity is an independent risk factor for hypertension and they are often found together^11^. A significant portion of our study subjects was obese which may enhance hypertension and cardiovascular risk and related mortality. A high prevalence of overweight and prehypertension was also found among our participants (51.6% and 39.7%, respectively). The overweight and prehypertension are also public health concerns worldwide as they contribute to the development of obesity and hypertension in later life. Therefore, controlling normal BMI and blood pressure, eating a healthy diet and regular physical exercise can be effective in reducing the risk of obesity and hypertension among our participants. Finally, academic staff are an important population group of the nation; therefore, it is recommended to control obesity and hypertension to prevent cardiovascular diseases and related health consequences.

The main strengths of our study were that both genders were included in the study and we analyzed most of the demographic, anthropometric and lifestyle-related data. However, there were some limitations to our study. First, we measured obesity and blood pressure obesity data on a single day, therefore, a causal relationship could not be determined. Longitudinal studies are needed to identify all types of risk factors for obesity and hypertension among the academic staff. However, many of the similar did not measure blood pressure and obesity data several times. Second, our sample size was relatively small, therefore, the present data do represent the entire academic staff in Bangladesh. Despite the limitations, the findings of the present study could be a basis for obesity and hypertension control in academic staff in Bangladesh.

## Conclusions

The prevalence of obesity and hypertension was higher among university academic staff members in Bangladesh. Our results showed that about 1 in 4, 1 in 2 and 1 in 3 of the academic staff were generally obese, abdominal obese and hypertensive, respectively. The prevalence of obesity was higher in female staff; whereas, hypertension was higher in male staff. Our study identified several risk factors that were associated with obesity and hypertension. The female gender and inadequate physical activity were independent risk factors for general and abdominal obesity. In contrast, age, BMI, WC, smoking and the presence of diabetes were the independent risk factors for hypertension. Our findings suggest that there is a need for comprehensive programs targeted at high-risk population groups such as females for obesity and males for hypertension. The comprehensive programs should focus on a healthy lifestyle, routine measurement of blood pressure, early diagnosis and treatment of the disease and increasing awareness to control and prevent obesity and hypertension among the academic staff in Bangladesh.

## Data Availability

The datasets used and/or analysed during the current study are available from the corresponding author upon reasonable request.
